# Impact of mhGAP-IG training on primary care physicians’ knowledge of mental, neurological and substance use disorders in Iraq

**DOI:** 10.1192/bji.2023.36

**Published:** 2024-02

**Authors:** Mohammed Al-Uzri, Hasanen Al-Taiar, Emad Abdulrazaq Abdulghani, Yassen Ahmed Abbas, Muhammad Suleman

**Affiliations:** 1 Consultant Psychiatrist, Assertive Outreach Service, Leicestershire Partnership NHS Trust, OSL House, Leicester, UK; 2 Consultant Psychiatrist, Oxford Clinic, Littlemore Mental Health Centre, Oxford, UK; 3 National Advisor on Mental Health, Iraqi Ministry of Health, Baghdad, Iraq; 4 President, Iraqi Red Crescent Society, Baghdad, Iraq; 5 Specialty Trainee in Psychiatry, Bradgate Mental Health Unit, Leicestershire Partnership NHS Trust, Leicester, UK. Email: drsuleman.psych@gmail.com

**Keywords:** Low- and middle-income countries, primary care, transcultural psychiatry, mhGAP, Iraq

## Abstract

To address the growing need for good-quality mental health service provision to patients in Iraq, mhGAP-IG 2.0 training in mental, neurological and substance use (MNS) disorders was delivered for primary care physicians in May–June 2022 by the Royal College of Psychiatrists (RCPsych) volunteers scheme. An innovative hybrid model was used to deliver this training to improve engagement compared with virtual training alone. Pre- and post-training assessment tools showed a significant improvement in participants knowledge of MNS disorders. Follow-up fortnightly supervision sessions by RCPsych volunteers were planned to help participants consolidate their learning in managing MNS disorders.

Mental, neurological and substance use (MNS) disorders are prevalent in all regions of the world and are major contributors to morbidity and premature mortality.^1^ In 2019, prior to the COVID-19 pandemic, an estimated 970 million people worldwide had a mental disorder, 82% of whom lived in low-and-middle-income countries (LMICs).^2^

Globally, current mental health systems are not meeting their populations’ needs. The gaps between prevalence and treatment vary across countries and from one mental disorder to another. Primary care can offer treatment of MNS disorders as it enables patients to get easier and faster access to services.^3^

In 2008, the World Health Organization (WHO) developed the Mental Health Gap Action Programme (mhGAP), to facilitate the scaling up of care, including diagnosis and management of MNS disorders in non-specialised healthcare settings in LMICs.^4^ The mhGAP Intervention Guide (mhGAP-IG) was developed in 2010 to assist in implementation of mhGAP. A revised version 2.0 was released in 2015.^5^

The global literature suggests a substantial impact of mhGAP-IG training on primary care physicians’ overall awareness and knowledge of MNS disorders. However, only a handful of these studies were quantitative.^6–8^ For example, Hughes et al (2016) reported that in-person training of 52 participants, 25 of whom were primary care physicians, with country-specific and needs-led mhGAP-IG training, showed an 8.5% improvement in post-training test scores.^8^

The Iraqi Red Crescent Society (IRCS) approached the Royal College of Psychiatrists (RCPsych) to help with a programme to improve primary care physicians’ mental health knowledge and skills. In response, the RCPsych volunteer scheme undertook to deliver a 5-day hybrid (in person and virtual) model of mhGAP-IG training to a group of primary care physicians in Iraq. This study aims to assess the impact of this training in improving the physicians’ knowledge of assessment and management of MNS conditions.

## Method

A team from the RCPsych volunteer scheme delivered the WHO mhGAP-IG 2.0 training to primary care physicians in Iraq between 28 May to 3 June 2022.

The training team comprised three consultant psychiatrists, two of whom (M.A.-U. and H.A-T.) attended personally, and a third consultant supported virtually from the Royal College of Psychiatrists, London. In addition, the National Advisor for Mental Health (E.A.A.) in the Ministry of Health, Iraq, joined the team and was supported by a local psychiatrist.

This was a hybrid model in which a subject matter expert from the UK delivered the bulk of the mhGAP training package online. This was aided by two consultant psychiatrists, trained in mhGAP, who facilitated the delivery of training on-site. This innovative approach ensured better communication between the online subject matter expert and the attendees and was supplemented by an interactive component of face-to-face teaching.

The 17 participants (11 male and 6 female) came from different specialties, such as paediatrics, obstetrics and gynaecology and orthopaedics, and had varying levels of expertise and knowledge. They worked as primary care physicians and provided weekly sessions with the Iraqi Red Crescent Society (IRCS) in mobile clinics. These mobile clinics provided care, including medicine, to the disadvantaged and displaced population across Iraq. The participants reported that they face a variety of mental health problems in their clinics but they have no formal training in mental healthcare and have limited knowledge of how to deal with these patients at the primary care level.

The participants were given a pre-training assessment, which consisted of 25 questions from the mhGAP-IG 2.0 training questionnaire^9^ modified to adapt to the local context and the doctors’ experience (see Supplementary Appendix, available at https://dx.doi.org/10.1192/bji.2023.36). The questions tested knowledge about MNS conditions and required either a ‘true/false’ decision or a choice of option in MCQs. Participants then received a 5-day training course using mhGAP-IG 2.0 manual. The training consisted of lectures and workshops in addition to small-group teaching, problem-based learning and role-play. The participants then answered the 25 questions again in a post-training assessment and the results were compared.

Informed consent was obtained from all participants. Ethical approval was not needed as this was a survey of participants in a training event (no patient involvement). However, the whole project was approved by the Iraqi Red Crescent Society.

## Results

The mean scores of the 17 primary care physicians in the pre-training and post-training assessments were 10.84/25 and 13.56/25 respectively (Table 1). This shows that there was an overall 25% improvement in knowledge about MNS disorders following mhGAP-IG training. Out of the 25 assessment questions, 17 were related to mental disorders and these showed an improvement in mean post-training score of 26.01%. The 7 questions that were related to neurological disorders showed the least improvement in mean score (11.54%), whereas the 3 questions about substance use disorders showed the most improvement (70%). The mode of the scores improved from 10 to 17. Mode and range were calculated only for the total score pre- and post-training and not for the subsections, because of the small sample size.

**Table 1:** Pre-training and post-training assessment scores for the 17 participants

Disorder	Assessment questions, *n*	Pre-training scores, mean (mode; range)	Post-training scores, mean (mode; range)	Improvement, %
Mental health	15	11.53	14.53	26.01
Neurological	7	11.14	12.43	11.54
Substance use	3	6.67	11.33	70
Total	25	10.84 (10; 1–16)	13.56 (17; 5–17)	25

## Discussion

Many doctors in Iraq face multiple challenges with their professional development and training opportunities. These include challenges related to access to regular supervision, which affect their ability to provide standardised care. Furthermore, unstable geopolitical situations can often influence the quality of and access to up-to-date medical education and training. This is true for all healthcare specialties but is particularly significant in mental health because there are only about 2 mental health professionals (psychiatrists, mental health nurses, psychologists and social workers) per 100 000 population.^10^ It is therefore imperative that the mhGAP-IG, a standardised training tool developed by WHO for global use, should not only be used to scale up Iraq's mental health services but should also be modified and adapted to accommodate the local sociocultural context.^11^

### Key findings

The mhGAP-IG training delivered in this study in Iraq proved successful in improving primary care physicians’ overall knowledge of MNS disorders, which is consistent with the literature and previous experience. However, although the increase in mean score is significant (25%), the average post-training score itself remains modest (13.56 out of 25).

There is evidence that case studies and simulations are classroom techniques associated with transformative learning.^12^ During the 5 days of this hybrid training model, doctors were able to review and discuss a variety of real-life clinical scenarios and benefited from the trainers in the room as well as enhanced communication and feedback with the subject matter expert online. This approach merged the benefit of ease of accessibility to online training with the interactive element of face-to-face training. Examples of transformative learning were various and included conducting a thorough risk assessment and interviewing psychiatric patients, which was challenging for some participants and took them out of their ‘comfort zone’. It is relatively safe to say that this cohort of doctors began to question their preexisting beliefs and concepts about mental illness. The feedback of primary care doctors during the training they had in May–June 2022 was very positive, with strong engagement and participation in the workshop.

There is also clear evidence that the participating doctors have improved in terms of their confidence in dealing with psychiatric patients and their ability to diagnose psychiatric patients and signpost them to the right support channel, such as seeing a specialist, starting medication or considering counselling.

### Implications and future plans

The use of a hybrid model for delivery proved to be successful as it addressed the limitation of online training by providing in-person facilitation while maintaining online expert input. In addition, linking trained staff to the local specialised services can provide a cohesive care pathway for patients with mental health needs.

However, the modest average post-training score on overall knowledge of MNS disorders indicates the need for more learning, which could be provided through supervision and case-based discussion. Furthermore, it is widely accepted that improvement following mhGAP-IG training is not sustainable without follow-up and supervision. Therefore, in line with literature,^6^ it was agreed that the participants will have regular supervision sessions. These sessions, which started in September 2022, are delivered every fortnight by volunteers from the RCPsych who are of Iraqi origin, to reduce cultural and language barriers. The primary care physicians bring cases from their clinics and use them for discussion in the sessions, offering an opportunity to share learning and consolidate knowledge. The ultimate objective for this cohort is to deliver better care for patients with mental health problems and to become mhGAP trainers.

It is also important that mhGAP-trained primary care physicians have access to more specialised mental health services when needed. Therefore, the office of the National Advisor for Mental Health provided details of mental health offices in the governorate to which the doctors can refer patients who need specialised psychiatric care. Integrating training and development into local services was an important step because it addresses the criticism of international work being delivered in isolation without linking with local services.

## Supporting information

Al-Uzri et al. supplementary material Al-Uzri et al. supplementary material

## Data Availability

The data that support the findings of this study are available from the corresponding author, M.S., upon reasonable request.
