# Supplementary material for: Impact of mhGAP-IG training on primary care physicians’ knowledge of mental, neurological and substance use disorders in Iraq
Source: BJPsych Int. 2024 Feb;21(1):14–6. doi: 10.1192/bji.2023.36 (PMC10803792; doi:10.1192/bji.2023.36)
Supplement: Al-Uzri et al. supplementary material [file S2056474023000363sup001.docx]

**Appendix A**

Pre and Post-training Test for mhGAP Base Course

Duration of test: 30 min

Same test is conducted before and after the mhGAP base course training

1. Put ✔ in the correct column.

|  | True | False |
| --- | --- | --- |
| 1. People with mental disorder usually cannot make decisions concerning their health |  |  |
| 1. People with mental disorder are best cared for in mental hospitals |  |  |
| 1. All people with depression should be treated with antidepressants |  |  |
| 1. Dementia is a normal part of aging |  |  |
| 1. Providing brief advice to people who have alcohol problems is effective |  |  |
| 1. Mental disorders are common in children and adolescents |  |  |
| 1. To stop acute seizures, diazepam by intramuscular route is the treatment of choice |  |  |
| 1. Severe chronic depression in a mother may lead to developmental delay in her children |  |  |
| 1. If a child shows over activity and inattention, then medication is usually needed |  |  |
| 1. Vitamin injections should be routinely used for somatic complaints with organic cause |  |  |
| 1. Asking people about suicidal thoughts increase the likelihood of suicide |  |  |

1. Put ✔ for the correct answer. There is only one correct answer for each question.
2. Which one of the following statements concerning depression ins correct?
3. Depression often presents with vague physical pain and fatigue.
4. Depression often presents with delusions and hallucinations.
5. Depression often presents with confusion.
6. Concerning antidepressants which of the following is correct?
7. The treatment should be continued even if the person suddenly becomes manic.
8. The treatment should be continued for 2-3 months.
9. The treatment should usually only be offered if the depression affects the person’s daily functioning.
10. Which of the following messages should be given to a person with depression?
11. Try to reduce your physical activity as much as possible.
12. Try to participate in social activities as much as possible.
13. Try to sleep as much as possible.
14. A 22-year-old girl says that she hears voices that no one else can hear and is convinced that someone wants to hurt her. Which of the following disorders is most likely present?
15. Psychosis
16. Depression
17. Mania
18. Concerning the management of acute psychosis
19. Medicines by injection will be required for most cases.
20. The person needs to be followed up at frequent interval.
21. The person should always be restricted (e.g., chained)
22. Concerning epilepsy, which of the following is correct?
23. For making diagnosis of epilepsy, first step is to do electroencephalography (EEG).
24. Two seizures in the last year are reason enough to start antiepileptic medicine.
25. Once the diagnosis of epilepsy is made in a woman with epilepsy, she should not marry or have children.
26. Concerning antiepileptic medications, which of the following is correct?
    1. Antiepileptic medication should be started at the maximum dose and then decreased.
    2. Antiepileptic medications should be combined for faster treatment.
    3. Antiepileptic medication can be stopped two years after the last epileptic seizure.
27. After a suicide attempt
    1. Leave the person alone resting in a quiet room.
    2. Restrain visits from family and friends.
    3. Remove means of self-harm.
28. Which of the following statement is correct concerning alcohol use?
    1. If people drink alcohol every day of the week, they are alcohol dependant.
    2. Alcohol use cannot cause seizures.
    3. People can have an alcohol problem even if they only drink once in one month.
29. Concerning people with dementia, which of the following is correct?
    1. People with dementia should be admitted to an institution as early as possible.
    2. It is critical to assess the carer’s (e.g., family member’s) strain and to support them.
    3. There is not much you can do to improve the symptoms and the living situation.
30. Concerning the management of child with developmental delay which of the following is correct?
    1. The child should not be allowed to attend a normal school.
    2. Medication can reverse the situation.
    3. Explain to the family that the child can learn new skills.
31. Concerning management of an adolescent with persistent aggressive and disobedient behaviour, which of the following is correct?
    1. Provide advice to the family and teacher.
    2. Punishment for unwanted behaviour is the best method to improve behaviour.
    3. Medication should be considered as soon as possible.
32. Concerning drug use disorder which of the following is correct?
    1. Imprisonment is the most effective intervention.
    2. Mothers who use drugs should not breastfeed.
    3. Discussing with the person their ideas about perceived benefits and potential harms of drug use is useful.
33. Which of the following statements concerning pharmacological treatment for people with mental disorder is correct?
    1. You usually do not need to obtain consent since the person does not understand.
    2. Antidepressants should only be given to adolescents after trying psychosocial treatment.
    3. Once the antipsychotic treatment starts, the person needs to continue taking the drug throughout life.
